# Therapeutic Potential of Human Adipose-Derived Stem Cells (ADSCs) from Cancer Patients: A Pilot Study

**DOI:** 10.1371/journal.pone.0113288

**Published:** 2014-11-20

**Authors:** Marta García-Contreras, César David Vera-Donoso, José Miguel Hernández-Andreu, José Manuel García-Verdugo, Elisa Oltra

**Affiliations:** 1 Facultad de Medicina, Universidad Católica de Valencia “San Vicente Mártir”, Valencia, Spain; 2 Servicio de Urología, Hospital Universitario y Politécnico La Fe, Valencia, Spain; 3 Laboratory of Comparative Neurobiology, Instituto Cavanilles de Biodiversidad y Biología Evolutiva, University of Valencia, CIBERNED, Paterna, Valencia, Spain; Faculty of Animal Sciences and Food Engineering, University of São Paulo, Pirassununga, SP, Brazil, Brazil

## Abstract

Mesenchymal stem cells from adipose tissue (ADSCs) are an important source of cells for regenerative medicine. The therapeutic effect of culture-expanded adipose derived stem cells has been shown; however, optimal xeno-free culture conditions remain to be determined. Cancer patients, specifically those undergoing invasive surgery, constitute a subgroup of patients who could benefit from autologous stem cell transplantation. Although regenerative potential of their ADSCs could be affected by the disease and/or treatment, we are not aware of any study that has evaluated the therapeutic potential of ADSCs isolated from cancer patients in reference to that of ADSCs derived from healthy subjects. Here we report that ADSCs isolated from subabdominal adipose tissue of patients with urological neoplasms yielded similar growth kinetics, presented equivalent mesenchymal surface markers and showed similar differentiation potential into distinct mesodermal cell lineages: adipocytes, chondroblasts and osteoblasts than ADSCs isolated from adipose tissue of age-matched non-oncogenic participants, all under xeno-free growth culture conditions. Molecular karyotyping of patient expanded ADSCs genomes showed no disease-related alterations indicating their safety. In addition, vesicles <100 nm identified as exosomes (EXOs) which may be at least partly responsible for the attributed therapeutic paracrine effects of the ADSCs were effectively isolated from ADSCs and showed equivalent miRNA content regardless they were derived from cancer patients or non-oncogenic participants indicating that the repair capabilities of xeno-free expanded ADSCs are not compromised by patient condition and therefore their xeno-free culture expanded ADSCs should be suitable for autologous stem cell transplantation in a clinical setting.

## Introduction

Human adipose tissue is an abundant and accessible source of multipotent mesenchymal stem cells (MSCs) which hold great potential for therapeutic applications in regenerative medicine and tissue engineering [1]–[3]. MSCs have been shown to be capable of differentiating into multiple cell types from the mesodermal lineage (osteoblasts, chondrocytes and adipocytes) but also into non-mesodermal lineage cells (neurons, cardiomyocytes and ectodermal skin) [4]–[7], reduce inflammation in damaged tissues, stimulate angiogenesis and reduce apoptosis [8]–[11]. MSCs role in modulation of tissue repair and immunomodulation has been attributed to their paracrine factor secretion potential, mitochondrial transfer and exosome (EXO) secretion capacity [12]–[14]. EXOs are secreted bilipid membrane vesicles of ∼50–100 nm with a complex cargo that are readily internalized by target cells inducing varied physiological effects [15]–[17].

Up to date different methods of isolation and expansion of MSCs have been applied. Most include animal derived products like fetal bovine serum which is a source of contaminating viruses, bacteria, mycoplasmas, prions and other pathogenic or toxic agents, and of xenogeneic antigens that might tipper undesirable immune responses [18], [19]. Xeno-free culture could enhance the safety and quality of transplanted *in vitro*-expanded stem cells [20], [21] and allow for the establishment of standard expansion methods, avoiding batch-to-batch variations. However their novelty and the big difference in price limit the number of studies that have used xeno-free medium until now.

Even though allogeneic infusion of *in vitro* expanded MSCs seems as a promising option for certain purposes [22]–[25] most of the ongoing or completed clinical trials up to date are based on autologous treatments [26]. In this sense, advanced age and disease state could limit individuals options, especially since the number and regenerative potential of MSCs seem to negatively correlate with age [27], [28] and population aging is at increase.

Urological cancer patients are good candidates for stem cell-based therapeutics. Stem cell therapies directed to promote healing after the invasive surgery procedures they often undergo or designed to lessen urinary incontinence, a common secondary problem associated to prostatectomy could result of benefit to them. Also, *in vitro* expanded autologous MSCs could be used for programmed organ remodeling surgeries or even complete organ engineering for transplantation. However, we are not aware of any study focused on the evaluation of the therapeutic potential and safety of urological cancer MSCs for autologous transplantation.

Here, we performed a comparative analysis of MSCs obtained from abdominal fat of urological cancer patients and non-oncogenic participants expanded *in vitro* under xeno-free conditions in an attempt to determine their suitability for autologous cell-based therapies using optimal conditions for the clinic. Characterization included cluster of differentiation (CD) expression patterns and morphology, growth kinetics, plasticity, EXOs release as a measure of MSCs paracrine therapeutic potential and safety.

## Materials and Methods

### Sample collection and ethics statement

This study was initiated upon approval by the local Ethics Committee of Hospital Universitario y Politécnico La Fe (Valencia, Spain). Freshly excised abdominal fat tissue was collected from patients undergoing neoplasic urologic surgical treatment or from non-oncogenic participants after the corresponding informed consent was signed.

### Subjects

All samples were waste materials collected as a side-product of surgery, from cancer patients (n = 5) undergoing elective surgical abdominoplasty procedures in the Department of Urology, Hospital Universitario y Politécnico La Fe, (Valencia, Spain) or non-oncogenic age-matched (±5 y) participants (n = 2) with a similar ethnic background (Table 1). Non-oncogenic participants corresponded to multiorgan donors. For all the described procedures ADSCs from both oncological and non-oncological patients, as, respectively, the test and control samples were obtained and analyzed, unless otherwise indicated.

**Table 1:** ADSC yields in number of cells (x 10^5^) obtained per gram of tissue.

Subject	Gender	Age	Yield (x10^5^)
**Patient 1**	Female	38	0,09524
**Patient 2**	Male	58	0,05714
**Patient 3**	Male	68	0,90476
**Patient 4**	Female	72	1,77339
**Patient 5**	Male	63	12,66968
**Non-oncogenic participant 1**	Male	41	0,36050
**Non-oncogenic participant 2**	Male	77	0,13232
	Mean±SD	59,57±15,02	0,23±0,46

### Isolation of ADSCs

Isolation of ADSCs was carried out using a mechanical and enzymatic method. Adipose tissue was transported and washed with saline solution NaCl 0,9% (B. Braun, cat. 12606097), fragmented with a surgical blade and digested with 1 mg/ml type I collagenase (Life technologies, cat. 17100-017), for 2 h at 37°C with shaking. The digested tissue was filtered through gauze to separate it from the undigested tissue and centrifuged at 500 g for 7 min at room temperature (RT). The supernatant (floating adipocytes) was discarded. The pellet (ADSC fraction) was resuspended in Hank's Balanced Salt Solution (HBSS +Ca +Mg) (Gibco, cat. 14025092) +1%BSA (Sigma, cat. A2153), filtered through a 140 µm nylon mesh and centrifuged at 500 g for 7 min at RT. Cells were then resuspended in erythrocyte lysis buffer on ice during 10 min and centrifuged again at 500 g for 7 min at 4°C. The isolated cells were then plated and expanded in culture medium. The culture medium was MesenCult-XF Medium (Stemcell technologies, cat. 05420) supplemented with 1% (v/v) penicillin/streptomycin (10000 U/ml penicillin, 10000 µg/ml streptomycin, Gibco cat.15140-122), and 2 mM L-glutamine (Gibco, cat. 25030-081).

### *In vitro* expansion and sampling of ADSCs

ADSCs were cultured at 37°C in a 5% CO_2_ air atmosphere with medium changes every 3 days. When the cells reached approximately 80% confluence, subculture (passage) was performed by disaggregation using MesenCult-ACF Dissociation Kit (Stemcell technologies, cat. 05426) (6 ml/flask) for 5 min, after a wash in phosphate buffer saline (PBS). Cells were counted and re-plated in a culture T-75 flask at a density of 250.000 cells. The dissociation solution was neutralized using the same volume of MesenCult-ACF Enzyme Inhibition Solution (Stemcell technologies, cat. 05428). Cell suspensions were centrifuged (500 g, 7 min) and the supernatants discarded. Pellets were resuspended in complete medium at indicated density. Counted was done in a Neubauer's chamber using Trypan Blue exclusion test for living cells (Sigma Aldrich, cat. T8154). Before each passage, cultures were photo-documented. Population doublings (PD) were calculated by using the formula *x* =  [log_10_ (NH) - log_10_(NI)]/log_10_(2), where NH is cell harvest number and NI inoculated number as previously described [29]. To obtain the cumulative population doubling, the population doubling for each passage was calculated and then added to the previous passage population doubling number. Cells then were subjected to further analyses, including ultra-morphology, differentiation potential, senescence and epitope analysis. Some aliquots were pelleted and stored in −80°C for RNA isolation.

### Flow cytometry

About 0,5–1×10^6^ cells were labeled with the following fluorescently labeled anti-human antibodies: CD73, CD90, CD105, CD34, CD11b, HLA ABC and HLA-DR (Table 2) and analyzed in different combinations. In summary cells were incubated for 30 min at RT and protected from light, washed (3X) with PBS centrifuging at 500 g at 4°C for 7 min. Washed cells were resuspended in 1 ml of PBS. Labeled samples were analyzed by flow fluorescence activated cell sorting analysis (FACS) (Beckman Coulter Cytomics, FC 500) at the specific fluorescence channels for each fluorochrome. Plots were generated using the CXP analysis software (Beckman Coulter).

**Table 2:** Mesenchymal stem cell surface markers.

Surface protein	Common name or description	Fluorochrome	Company	Catalog number	Dilution factor
**CD 11b**	Integrin alpha M chain	FITC	Beckman Coulter	IM0530	1∶9
**CD 34**	Sialomucin-like adhesion molecule	PC 5	Beckman Coulter	A07777	1∶10
**CD 73**	Ecto 5′ nucleotidase	PE	Miltenyi Biotec	130-095-182	1∶11
**CD 90**	Thy-1, T-cell surface glycoprotein	PC 5	Beckman Coulter	PN IM3703	1∶10
**CD 105 Clone 1G2**	SH-2, endoglin	-	Beckman Coulter	PJ IM1226	1∶10
**Goat anti-mouse IgG3**	IgG3	FITC	Beckman Coulture	732372	1∶10
**HLA ABC**	Major histocompatibility class I Antigen	FITC	Beckman Coulter	PN IM1838U	1∶5
**HLA DR**	Major histocompatibility class II antigen	PE	Beckman Coulter	IM1639	1∶5

Abbreviations: FITC, fluorescein isothiocyanate; IgG, immunoglobulin, PE, phycoerythrin.

### Adipogenic differentiation

For adipogenic differentiation, cells were cultivated in 6-well plates containing 2 ml of Mesencult-XF basal medium supplemented with adipogenic stimulatory supplements (MesenCult Adipogenic Stimulatory Supplements (Human), cat. 05403) for 15 days, under standard growing conditions (37°C, 5% CO_2_). Culture media was not changed unless medium became yellow/orange in color, in which case a half-medium change was performed. After the 15-day incubation period, the medium was removed and the cells were washed in PBS. Fixation with 4% paraformaldehyde (PFA) for 30 min was followed by two washes in distilled water and one of 60% isopropanol, each for 5 min at RT. Adipogenic differentiation was confirmed by Oil Red O staining (Sigma, cat**.** O0625) following manufacturer's instructions. In brief, cells were incubated with Oil Red O dye at RT for 10 min in 2 ml. Dye was carefully removed and the plate was washed four times with distilled water. Then, cells were observed using an optical microscope (Nikon Eclipse TE2000-U light inverted microscope, Nikon Inc, Melville, NY, USA) and photographed. Adipocytes were identified as cells with red-stained lipid vesicles [4], [20], [30].

### Osteogenic differentiation

Cells were cultured in six well plates containing 2 ml of MesenCult MSC Basal Medium (Human) (Stemcell technologies, cat. 05401) supplemented with Osteogenic Stimulatory Supplement (Stemcell technologies, cat. 05405), β-Glycerophosphate 1M (Stemcell technologies, cat. 05406), dexamethasone (Stemcell technologies, cat. 05407) and ascorbic acid (Stemcell technologies, cat. 07157) for 15 days (Osteogenic medium). The plates were kept in a humidified incubator at 37°C and 5% CO_2_ and the culture medium was changed every three days. After the 15-day period, the medium was removed and the cells were washed with PBS. After fixation of the cells with PFA 4%, for 30 min the cells were washed three times with distilled water. Alizarin red S staining (Sigma, cat. A5533) was used to confirm osteogenic differentiation using manufacturer's protocol. Briefly, the cells were incubated in 2 ml solution of sodium alizarin at RT for 30 min. The dye was carefully removed and extensive washing with distilled water followed. The fixed and dyed cells were observed using optical microscope (Nikon Eclipse TE2000-U inverted microscope, Nikon Inc, Melville, NY, USA) and photographed [20], [30].

### Chondrogenic differentation

For chondrogenic differentiation, the cells were cultured in six well plates containing 2 ml of complete medium supplemented with StemPro Chondrogenesis Differentiation Kit (Gibco, cat. A10071-01) for 15 days. Plates were kept in a humidified incubator at 37°C and 5% CO_2_ and culture medium was changed every three days. After the 15-day period, the differentiation medium was removed and the cells were washed with PBS. Then, fixed with 2% glutaraldehyde for 20 min and washed three times with PBS. The cells were then incubated in Alcian blue 8GX solution (Acros organics, cat. 400460100) overnight at RT. The dye was carefully removed and the plate was washed three times with 0.1M HCl and twice with PBS. After fixation and staining, cells were observed using an optical microscope (Nikon Eclipse TE2000-U inverted microscope, Nikon Inc, Melville, NY, USA) and photographed [4], [20], [30].

### Senescence associated β-galactosidase staining

pH-dependent senescence associated with β-galactosidase activity was analyzed using the SA-β-gal staining kit (Cell Signaling Technology, cat. 9860) according to manufacturer's protocol. In brief, cells were incubated in freshly prepared senescence-associated β-galactosidase blue stain solution (X-gal) overnight at 37°C. On the following morning cells were observed and images taken under an inverted light microscope Nikon Eclipse TE2000-U (Nikon Inc, Melville, NY, USA).

### RT-PCR

Total RNA was extracted from cultured cells with the mirVana miRNA isolation Kit (Ambion, cat. AM1560) according to manufacturer's protocol. RNA yields and purity were determined by the 260/280 and 260/230 nm ratios with a Thermo Scientific NanoDrop ND-2000 (Thermo Scientific, Wilmington, USA). cDNA was prepared using 100–200 ng of total RNA and M-MuLV reverse transcriptase (New England Biolabs, cat. EP0352) by oligo-dT priming. The cDNA was amplified by PCR using the ABI GeneAmp PCR System 2400 (Perkin Elmer, Applied Biosystems, Boston, MA) and the following cycling conditions: 94°C for 30 s, 54-60°C (depending on primer Tm) 30 s, and 72°C for 30 s (35 cycles), after a single initial denaturation cycle at 94°C for 5 min. PCR primer sets are shown in Table 3. PCR products were separated on a 2% agarose gel and visualized with realsafe stain.

**Table 3:** List of primers for RT-PCR.

Gene name	Amplicon lenght (bp)	Forward primer	Tm(°C)	Reverse primer	Tm(°C)
**Atg 5**	105	TGATCCTGAAGATGGGGAAA	53,3	TCCGGGTAGCTCAGATGTTC	56,3
**Atg 7**	90	CGGCGGCAAGAAATAATG	52,0	CCCAACATCCAAGGCACTAC	55,9
**Beclin 1**	160	GAGGGATGGAAGGGTCTA	52,7	GCCTGGGCTGTGGTAAGT	57,7
**GAPDH**	180	TGAAGGTCGGAGTCAACGGAT	61,1	TTCTCAGCCTTGACGGTGCCA	61,4
**h18S rRNA**	151	GTAACCCGTTGAACCCCATT	58.09	CCATCCAATCGGTAGTAGCG	57.9
**β Actin**	282	ATATCGCCGCGCTCGTCGTC	65,38	GAGCCACACGCAGCTCATTG	65,2
**Nodal**	428	GCAGCTGTCCAGCCCTGTGG	65,83	GTCGACGGTGCCTCTTGCCC	65,8
**UTF1**	520	CAAGTTTCGCGAGGCGCACG	65,38	CAGCGGGCCCAGGATGTTCG	65,9
**ABCG2**	366	TCTTCTCCATTCATCAGCCTC	57,17	TCTTCTTCTTCTTCTCACCCC	56,6
**CD29**	598	GCGCGTGCAGGTGCAATGAA	65,12	TCAGGTTGGACCGGCTGGGG	66,4
**CD44**	324	AAGACATCTACCCCAGCAAC	57,21	CCAAGATGATCAGCCATTCTGG	59,1

### qRT-PCR

Total RNA was isolated from ADSCs and Exosomes with the mirVana miRNA isolation Kit (Ambion, cat. AM1560) using manufacturer's protocol. Reverse-transcription was performed using miScript II RT Kit (Qiagen, cat. 218161) according to manufacturer's guidelines. cDNAs were used for Real time PCR using miScript SYBR Green PCR kit (Qiagen, cat. 218073). The sequences of the forward primers used are shown in Table 4.

**Table 4:** List of primers for qRT-PCR.

Target name	Sequence	Tm(°C)
**Let-7**	AAACCGTTACCATTACTGAG	47,4
**miR-21**	TAGCTTATCAGACTGATGTT	47,9
**miR-1260b**	ATCCCACCACTGCCACC	57,9
**miR-1908**	CGGCGGGGACGGCGATTGG	66,9
**miR-143**	TGAGATGAAGCACTGTAGC	51,9
**miR-145**	GTCCAGTTTTCCCAGGAATCC	55,7
**miR-338**	TCCAGCATCAGTGATTTTGT	52,1
**miR-451**	AAACCGTTACCATTACTGAG	49,8

### Exosome isolation

EXOs were purified from xeno-free cell culture supernatants of adipose derived stem cells by ultracentrifugation. Supernatant fractions collected from ADSC cultures were centrifuged at 300 g for 10 min, 2,000 g for 20 min and 10,000 g for 20 min to eliminate large dead cells and debris. After, filtration through 0.22-µm filters to eliminate impurities, the final supernatant was ultracentrifuged at 110,000 g for 70 min to pellet EXOs. Recovered EXOs were washed with PBS and centrifuged again at 110,000 g for 70 min. The pellet was resuspended in 50 µl of PBS to obtain a concentrated EXO fraction.

### Western blot analysis

ADSCs and EXOs were lysed in Tissue Protein Extraction Reagent (T-PER) (Thermo Scientific, cat. 78510) following manufacturer recommendations. Protein concentrations were measured by the Bradford method (Thermo Scientific Pierce Coomassie (Bradford) Protein Assay, cat. PI-23200). Samples were mixed with non-reducing Tris-glycine SDS sample buffer, then heated 95°C for 5 min and loaded onto a 10% SDS-polyacrylamide gel. The gel was run under denaturing conditions at 80 V for 2 h then transferred to a PVDF membrane (GE Healthcare Life Sciences, cat. 0485288). After transfer, membranes were blocked in 3% non-fat milk PBS for 2 h and incubated at RT for 2 h with rabbit-anti CD63 (Abgent, cat. AP5333b). Following washing in PBS-T, the membranes were incubated for 1 h with goat-anti-rabbit HRP-linked secondary antibody (Santacruz, cat. sc-2004) and developed with Luminata Forte Western HRP substrate (Millipore, cat. WBLUF0500) using an ImageQuant LAS 4000 chemiluminesce detector system.

### Electron microscopy

For ultrastructural analysis, cells were grown on permanox coverslips (8 well chamber slides) at a density of 3.5×10^3^ cells/chamber. Chambers were kept in a humidified incubator (37°C, 5% CO_2_) and culture medium was changed every three days until sub confluence. Cells were then washed three times with freshly prepared medium, once with PBS and then fixed with 3.5% glutaraldehyde for 30 min at 37°C. The fixative solution was removed and the cells were washed twice with PBS. Cells were postfixed in 2% OsO_4_ for 30 minutes at room temperature and stained in 2% uranyl acetate in the dark for 1 h at 4°C. Finally, cells were dehydrated in ethanol, rinsed with propylene oxide (Lab Baker, Deventry, Holland) and embedded overnight in Araldite (Durcupan, Fluka, Buchs SG, Switzerland). Upon polymerization, embedded cultures were detached from the chamber slide and glued (Super glue, Loctite) to Araldite blocks. Semi-thin sections (1.5 µm) were cut with an ultramicrotome (Ultracut UC-6, Leica, Heidelberg, Germany) mounted onto slides and stained with 1% toloudine blue. Ultrathin sections (70 nm) were prepared with the ultramicrotome and stained with lead citrate. Images were obtained with a transmission electron microscope (FEI Tecnai Spirit G2, Eindhoven, The Netherlands), using a digital camera (Morada, Soft Imaging System, Olympus).

Purified EXOs were fixed with 1% glutaraldehyde in PBS. After rinsing, a 20 µL drop of the suspension was loaded onto a formvar/carbon-coated grid, negatively stained with 3% (w/v) aqueous phosphotungstic acid for 1 min, and observed by transmission electron microscopy (TEM) in a Tecnai Spirit G-2 apparatus; (FEI, Eindhoven, The Netherlands).

### Molecular karyotype

Affymetrix CytoScan750 arrays were used to evaluate copy number gains and losses of heterozygosity (LOH) in ADSCs samples of patients and non-oncogenic participants. These arrays contain more than 2.6 million copy number markers of which 750,000 are “genotype–able” SNPs and 1.9 million are non–polymorphic probes. DNA was extracted from ADSCs for each of the two oncological patient samples analyzed, by using a QIAamp DNA Mini Kit (Qiagen, cat. 51306), following manufacturer's instructions. DNA quantity and quality was measured with spectrophotometer NanoDrop ND-2000 (Thermo Scientific) by absorbance ratios at 260/280 nm. The integrity of total DNA was measured by 0,8% agarose gel electrophoresis. Copy number and genotyping analyses were performed using Affymetrix Chromosome Analysis Suite (ChAS) software at the Array Service, Genomic Unit IIS La Fe.

### Statistical analysis

Data were analyzed by the unpaired 2-tailed Student's *t* test or Multiple t test, as indicated. The Holm-Sidak method correction was used to determine the significance of differences in multiple comparisons. Data corresponded to means ±SD of at least three independent replicates. All statistical analyses were performed using GraphPad Prism 6 (GraphPad Software, Inc., La Jolla, USA); values with *p*<0.05 were considered statistically significant.

## Results

### Population and cell yields

Patient's mean age was 60, range between 38 and 72 years, (n = 5), and non-oncogenic participants mean age was 59, range 41 to 77, (n = 2) (Table 1). Patients suffered from renal, bladder or prostate cancers and healthy tissue came from donors that suffered accidental traumatic death.

Average adipose-derived stem cell yield following initial expansion was (2,28±4,62) ×10^5^ and (3,10±5,4) ×10^5^ cells per gram of tissue from either patient or non-oncogenic participants, respectively. Yields were highly variable with a tendency to obtain lower yields from larger pieces of tissue (Table 1).

### Growth potential and senescence

**Figure 1:**
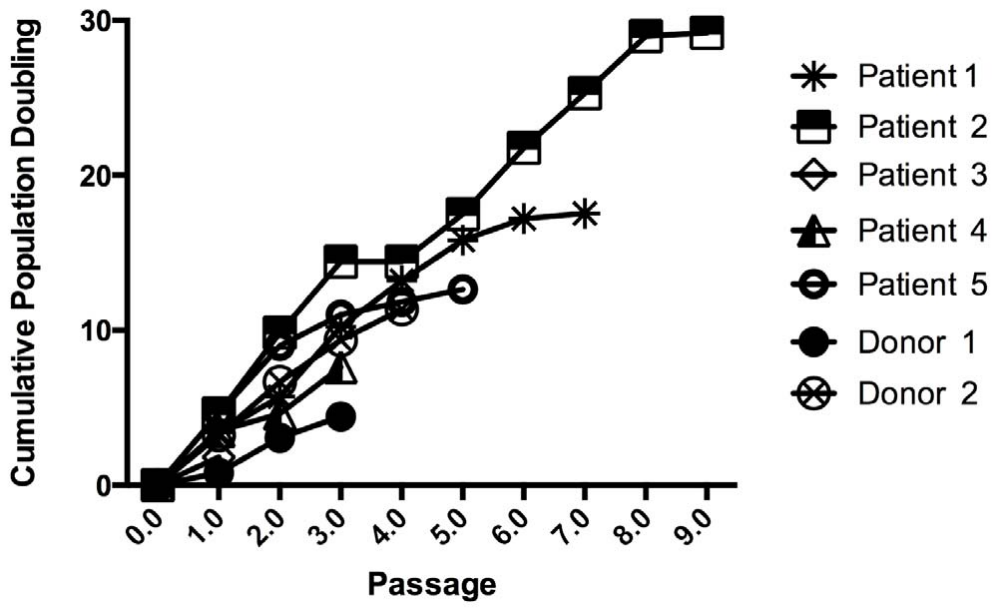
Growth kinetics of ADSCs. Results are presented as cumulative populating doublings of ADSCs derived from five different cancer patients and two non-tumorigenic participants (donors) under xeno-free culture conditions. Cell numbers were determined at the end of every passage and cumulative population doublings were calculated as described in Materials and Methods.

To investigate cell growth potential of cells from either group of participants, population doublings (PD) of each sample were determined at every passage up to passage 6, equivalent to day 58 in culture on average (Figure 1). The difference between patients (6.292±1.394) and donor (4.869±1.801) PDs was not significant (p>0.5), as determined by unpaired t-test analysis of the data. Semi-confluence at passage 1 varied about 3.6±1.2 PD for patients and 2.02±1.7 PD for non-oncogenic participants (Figure 1), coinciding with previous reports [31]–[33].

Although no abnormal growth behavior of cancer patient ADSCs was observed, it was important to determine whether these cells could present any growth advantages on longer culture periods. In order to evaluate expansion-time-related cell senescence, two of the analyzed samples, corresponding to patients 1 and 2 were further grown to late passages, specifically up to passage 10 and 8 respectively. Growth after passage 10 in culture was no longer exponential (Figure 1) indicating a limited expansion potential under the growth conditions used. Senescence was determined by changes in β-galactosidase activity of early *vs* late passages. As shown in Figure S1, β-galactosidase activity increased with passage. Most of the cells in early passages did not present evidence for the processing of the chromogenic X-gal substrate as cells from later passages did. This increase in β-gal staining between early and late passages (0.0390±0.0046 vs 0.1717±0.0181) was significant (p<0.005). This suggests that the replicative senescence of MSCs is a continuous time-dependent process as previously proposed [31], also for ADSCs obtained from cancer patients.

In addition, we evaluated the senescence-associated process of autophagy by RT-PCR analysis of autophagy-related genes. At late passages (passages 8–10) mRNA expression levels of Atg5 appeared upregulated (1.61±0.04 fold) while Atg7 were slightly inhibited (−1.10±0.03 fold) and Beclin1 levels showed a marked downregulation (−10.80±0.03) (Figure S1 C). The observed large accumulation of autophagosomes, at these late passages, evidenced by ultraestructural analysis of their cytoplasms (Figure S1 D, E) coincided with a reduction in growth rate suggesting that the increase in autophagy at late passages associates with replicative senescence, as previously reported [34]–[36]. The fact that ADSCs from patients senesce upon culture indicates that they are not tumorigenic.

### Morphology

**Figure 2:**
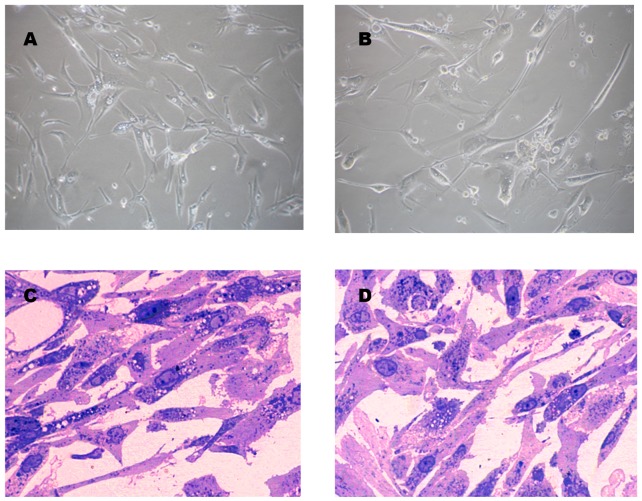
Morphology of *in vitro* expanded ADSCs. Representative phase-contrast images of *in vitro* expanded ADSCs from cancer patients (A) and non-tumorigenic participants (donors) (B) at passage 4, and toluidine blue staining of semithin sections of the same cells (C and D, respectively) (magnification 20X).

To examine possible morphological changes between non-oncogenic participants and neoplasic patient ADSCs at different passages, we evaluated their morphological and ultra-structural features by phase-contrast microscopy and transmission electron microscopy (TEM) at every passage up to passage 4, which is the recommended passage for therapy [37]. Images revealed all cells had spindle and multipolar shape morphology. No morphological difference between cells of either group of participants was observed (Figure 2 A, B).

Semi-thin sections did not show any appreciable morphological differences either, with most cells presenting a single nucleus (Figure 2 C, D). The nuclei contained one or two nucleoli and abundant nucleoplasm. Cells had intact membranes with pseudopodia structures on the surface and intact organelle structures. Rough endoplasmic reticulum (RE) and mitochondria were detected in both the inner and peripheral endoplasmic zones. Peripheral zones exhibited absence of organelles but contained vacuoles and vesicles.

**Figure 3:**
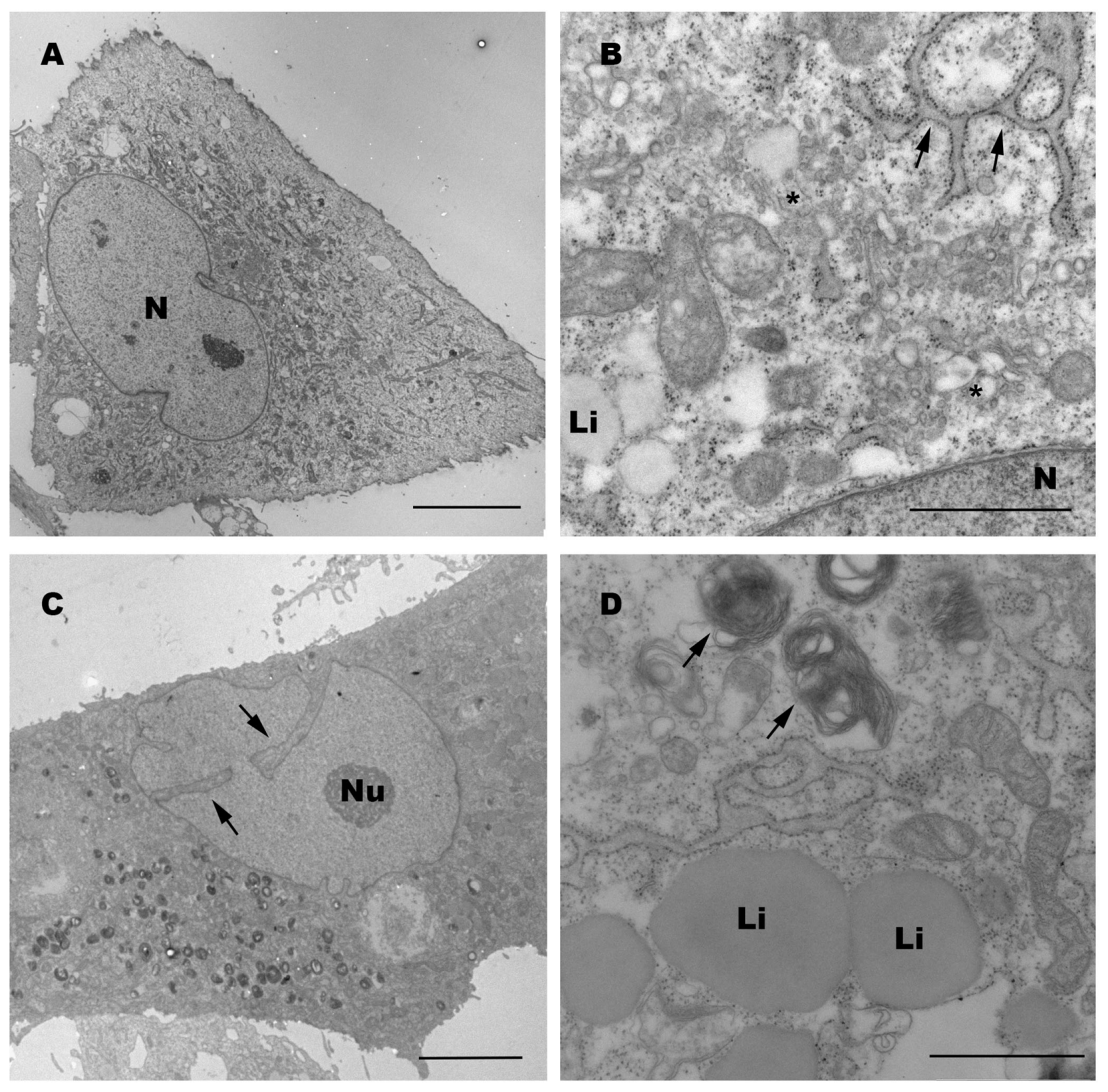
Transmission electron microscopy images of *in vitro* expanded ADSCs. ADSCs from cancer patients at passage 2 (A) and passage 4 (C) show cytoplasms enriched with organelles and large nucleus (N) with loosely packed chromatin. A detail of the ADSC from passage 2 (B) shows organelle enrichment, rough endoplasmic reticulum (arrows) and large Golgi cisternae (asterisk) with some lipid drops (Li). ADSCs at passage 4 (C) show prominent nuclear invaginations (arrows) and enlarged nucleoli (Nu). At low magnification also shows abundant electro-dense organelles. At a higher magnification abundant rough endoplasmic reticulum cisternae, lipid drops (Li) and abundant membranous structures or autophagosomes are appreciated (D). Scale bar 10 µm (A–C); 1 µm (B–D).

To increase resolution we obtained transmission electron microscopic images of either group of ADSCs. Cells at passage 2 showed abundant and enlarged rough RE, numerous Golgi cisternae and a large number of dictyosomes distributed along the cytoplasm that also contained mitochondria, lipid drops and abundant bundles of filaments (Figure 3 A, B). Electrodense bodies were also found next to dictyosomes.

The nuclei contained one or two nucleoli and presented loosely packed chromatin, sometimes showing deep invaginations and abundant nucleoplasm. At passage 4, cells showed some slight differences with respect to passage 2 including a higher tendency to invaginated nuclei, larger nucleolus and an increase in the number of bundles of filaments and in the number of electrodense membranous body-like structures or autofagosomes (Figure 3 C, D). However, no qualitative differences could be appreciated between groups, allowing us to conclude that ADSCs from cancer patients are identical to those of non-oncogenic participants at the morphological level.

### Immunophenotype

**Figure 4:**
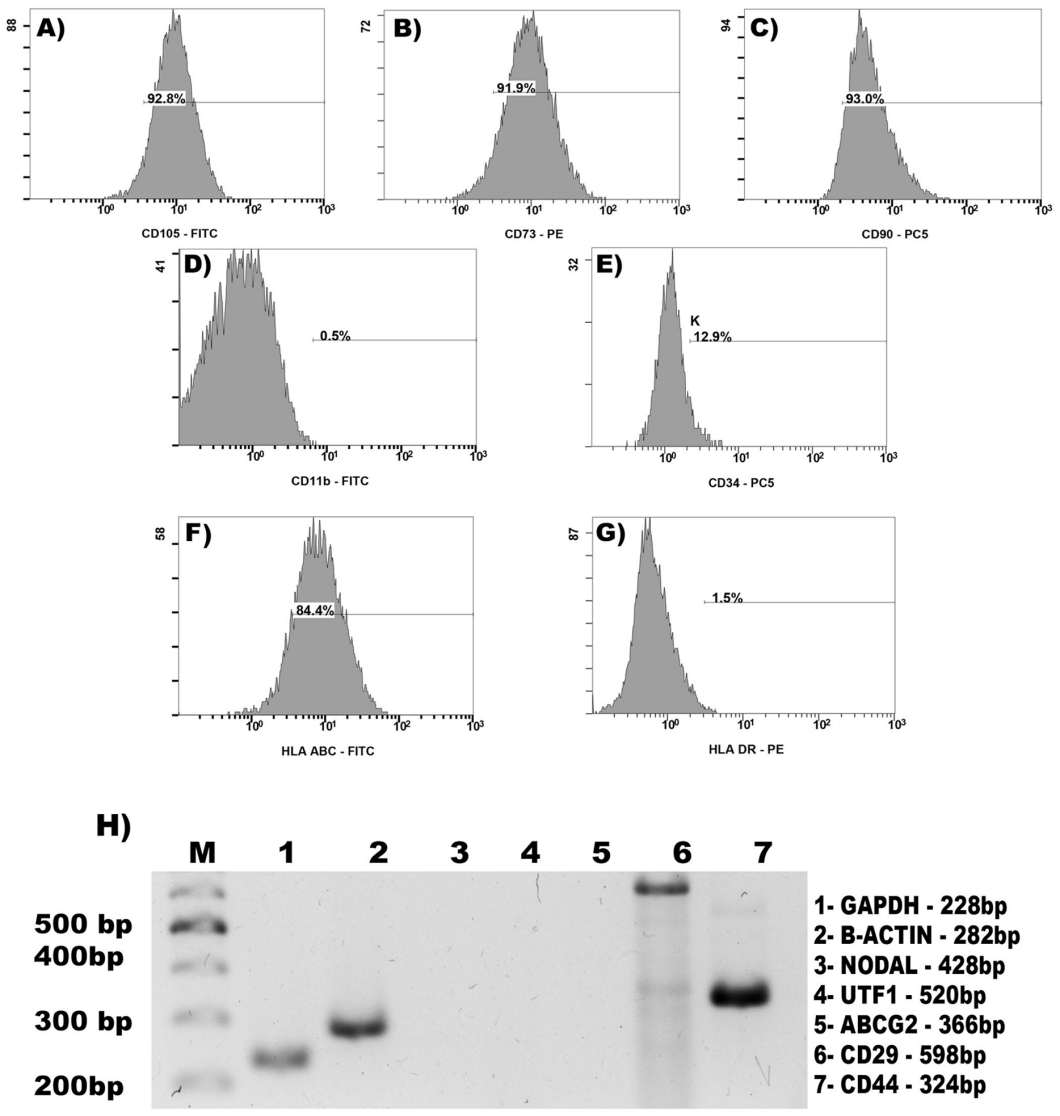
Expression of ADSC surface markers in cancer patient cells. Representative flow fluorescence activated cell sorting (FACS) and RT-PCR analysis of *in vitro* expanded ADSCs from cancer patients at passage 4. Cells were positive for CD105 (A), CD73 (B), CD90 (C), CD29 (H-6), CD44 (H-7) and HLA ABC (F) but do not express CD11b (D), NODAL (H-3), UTF1 (H-4), ABCG2 (H-5), or HLA DR (G); while CD34 was partially positive (E), as previously described. Lanes H-1 and H-2 show RT-PCR amplification of the house keeping genes GAPDH and β-actin, respectively.

In order to confirm that our expanded ADSCs complied with the minimal mesenchymal criteria established by the International Society for Cellular Therapy [38] we performed flow cytometric analysis of ADSCs from patients and non-oncogenic participants at passage 4 (Figure 4 A–G and Figure S4 A–F).

As expected, cells were positive for the mesenchymal CD73, CD90 and CD105 markers and negative for the hematopoietic marker CD11b. In addition they were positive for the histocompability antigen class I HLA-ABC but did not express HLA-DR surface molecules under an unstimulated state, as previously described [38]. Unexpectedly, CD34, a marker for hematopoietic and endothelial progenitors [39], was positive in 3-12% of the cells.

We also performed RT-PCR analysis for additional mesenchymal and pluripotency markers. The mesenchymal CD44 and CD29 markers were clearly positive while no amplification of the pluripotency NODAL and UTF 1 markers was obtained. We also failed to amplify the multidrug-resistance transport protein ABCG2 which is a pluripotency marker formerly associated with a subpopulation of MSCs with neurogenic potential [40], suggesting a possible limitation of these cells for the therapy of nervous tissue. The antigen surface profiles described were similar for MSCs from patients and non-oncogenic participants (data not shown).

### Cell plasticity

**Figure 5:**
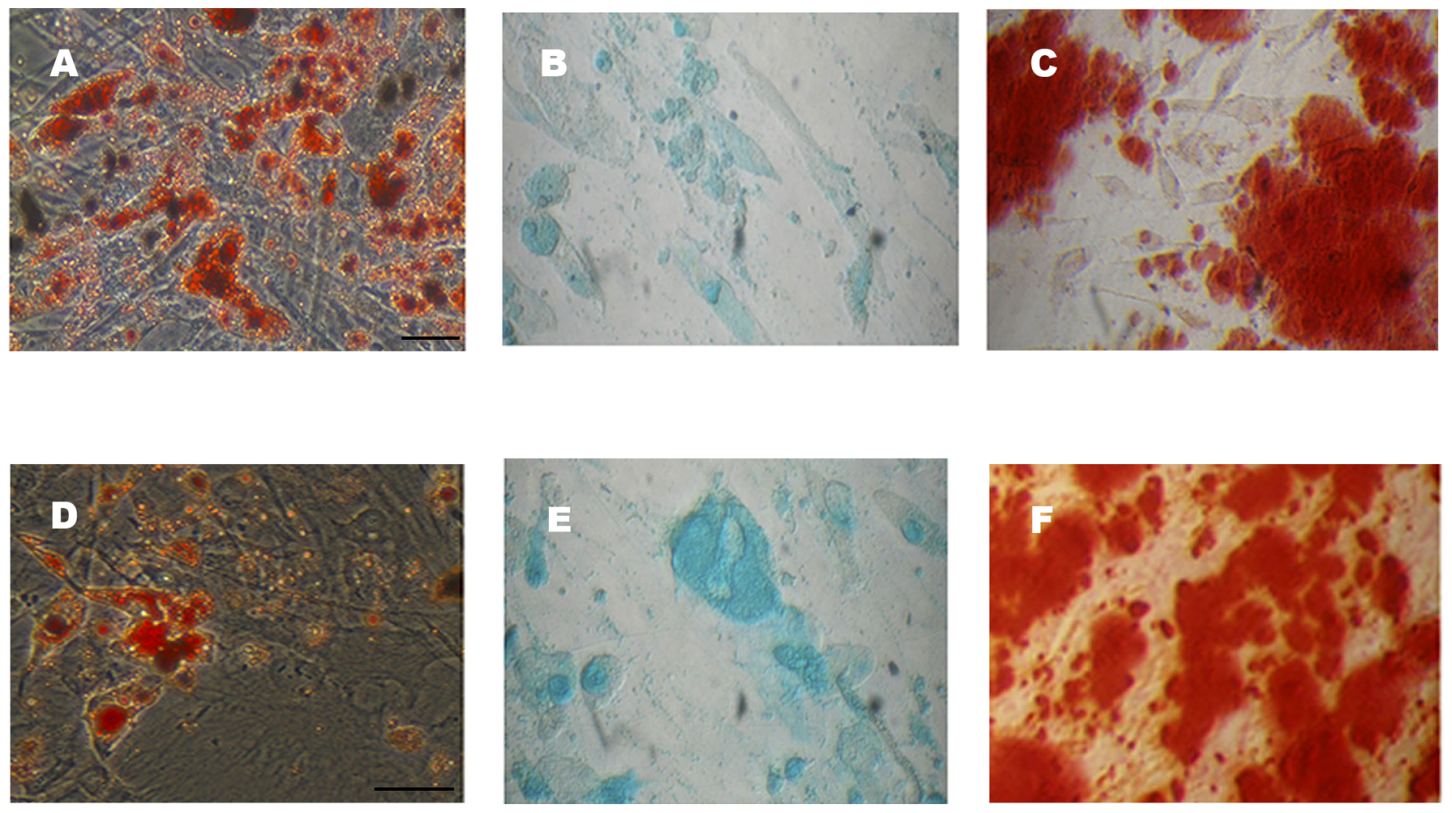
Differentiation potential of ADSCs from cancer patients and non-oncogenic participants. Representative microscopic images of differentiated *in vitro* expanded ADSCs from cancer patients (A–C) and non-oncogenic participants (D–F). Adipogenesis was evidenced by Oil Red-O staining (A, D); chondrogenesis by glycosaminoglycan alcian blue staining (B, E) and osteogenesis by alizarin red staining (C, F) after 15 days of differentiation induction (Scale bar  = 0,2 pixels).

The plasticity of the expanded ADSCs was determined by their differentiation potential. Cells at passage 4 from either patients or controls were cultured in specific differentiation media, as described in Materials and Methods. Cells in adipogenic media contained abundant vacuoles distributed along their cytoplasms as shown by Oil Red O staining (Figure 5 A, D), indicating that differentiation to adipocytes had occurred. Osteogenesis was evidenced by the presence of aggregates with nodule-like structures which were stained with Alizarin Red detecting mineral deposition (Figure 5 C, F). Chondrogenic differentiation was assessed using Alcian Blue staining that revealed high content of cartilage specific proteoglycans in the cultures (Figure 5 B, E). ADSCs from both patients (Figure 5 A–C), and non-oncogenic participants (Figure 5 D–F), were equally capable of efficiently differentiating into adipogenic, chondrogenic and osteogenic lineages indicating that ADSCs derived from our cancer patients possesses similar cell plasticity to those derived from non-oncogenic participants.

### Paracrine potential

**Figure 6:**
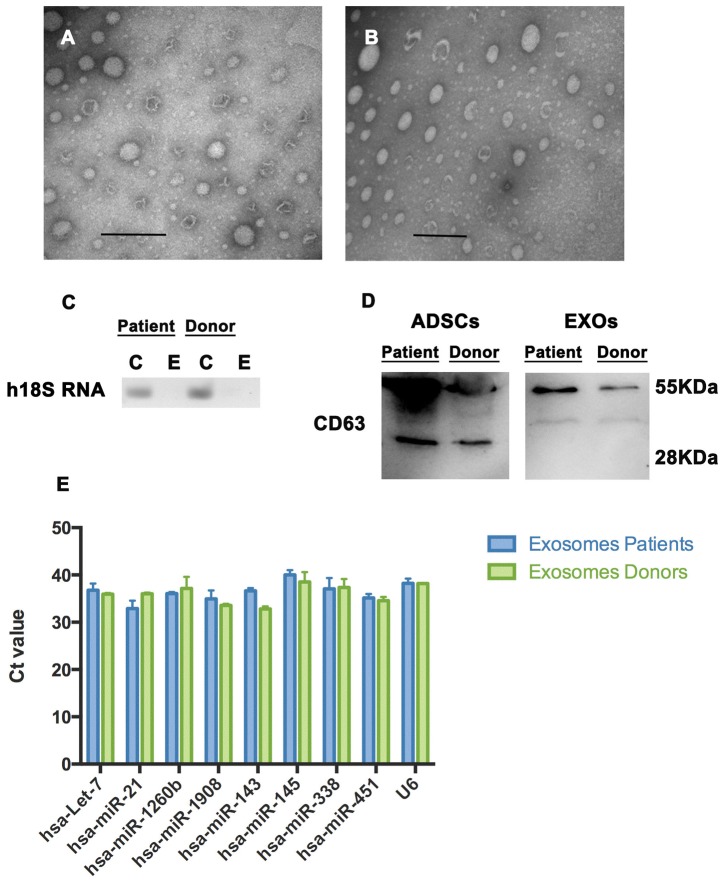
ADSC-derived Exosome isolation and characterization from cancer patients and non-oncogenic participants. Isolated microvesicles transmission electron microscopy images from cancer patients (A) and non-oncogenic participants (B) are shown. Lack of 18S RNA in EXOs was evidenced by RT-PCR analysis, total RNA from ADSCs was used as control (C). CD63 exosome marker evidenced by western blot analysis of total protein extracts prepared from microvesicle fractions and cell lysates (D). miRNA levels were determined by qRT-PCR analysis of total RNA prepared from EXOs; using U6 as an internal control (E).

Although MSC cell plasticity could be related to capacity of engraftment for the repair of damaged tissue, MSC therapeutic potential has repeatedly been attributed to their immunomodulatory and anti-inflammatory paracrine effects [11], [12], [41], [42]. In particular, cytokine/chemokine secretion, mitochondrial transfer and microvesicle (EXOs) secretion in response to injury has been widely reported [43]–[49]. EXOs have been proposed as paracrine effectors on the surrounding damaged tissue [14], [16], [50]. In addition to proteins and mRNAs, miRNAs have been shown to be directionally packaged in these vesicles [51], [52]. Since the molecular cargo of the EXOs could be related to MSCs paracrine potential, we proceeded to analyze miRNA content of EXOs isolated from ADSCs at passage 4 from both, patients and non-oncogenic participants. First, isolated EXOs were analyzed under an electronic transmission microscope to confirm that small vesicles with a <100 nm in size were present in the isolated fraction (Figure 6 A, B). The presence of the CD63 EXO marker was confirmed by western blot analysis of extracts prepared from the EXO fraction of both patients and non-oncogenic participants (Figure 6 D). EXOs, however, lacked 18S rRNA (Figure 6 C), coinciding with previous reports [52], [53].

T-test analysis of miRNA levels in ADSCs and their EXOs showed no significant differences (p>0.05) between some miRNAs (including miR-1908, and miR-338-3p) indicating that they are present at similar levels in both compartments: cells and vesicles, while other miRNAs (including let-7-a-1, miR-21 and miR-1260b) showed significant differences (p<0.05), suggesting that they preferentially pack into EXOs (Figure S2). This tendency, evidenced by real time RT-PCR analysis, was observed in both, patient and non-oncogenic participant samples.

Also, while some of the tested miRNAs had already been found in EXOs from ADSCs (let-7-a-1, miR-21, miR-143, miR145 and miR-451a) other have been identified here in ADSC-derived EXOs for the first time (Table S1). This is the case of miR-338-3p, miR-1260b and miR-1908.

When relative amounts of miRNAs in EXOs from patient and non-oncogenic participant ADSCs were compared we observed similar levels (no significant differences in t-test analysis, p>0.05) regardless of their origin (Figure 6 E) indicating no association between miRNA load and disease state of the participants, even for those miRNAs previously found in EXOs of cancer patients, as is the case of let-7-a-1, miR-21, miR145, miR-451a and miR-1908 [54].

### Safety

In addition to cancer-associated miRNA expression patterning as a measure of ADSC-derived EXOs safety, it is necessary to evaluate lack of genomic aberrations in the expanded ADSCs to ensure safe autologous cell-based treatments in cancer patients. For that purpose we performed molecular karyotyping of two of the patient ADSCs cells at passage 4 (Figure S3). The results failed to show cancer-associated alterations in their genomes indicating that no contaminating cancer cells were present in the cultures and that the culture conditions used did not induce transformation of the cells, at least up to passage 4. Thus, ADSCs derived from our participating patients, expanded under xeno-free culture conditions [21], [55] could be classified as safe for therapeutic purposes.

## Discussion

Patients suffering from urological neoplasias frequently suffer from health problems directly or indirectly related with cancer therapeutics that could be, at least partially solved by stem cell-based treatments, as for example bladder reconstruction or urinary incontinence. Characteristics of ADSCs from cancer patients might be the same as those of non-oncogenic participants, however little information on the characteristics of ADSCs from cancer patients is available. Due to the therapeutic potential applications of ADSCs in regenerative medicine for cancer patients, a focus on detailed characterization of autologous samples, including efficacy of isolation, growth rates, differentiation potential and safe expansion for clinical use was at need. To test whether *in vitro* expanded cultures of ADSCs from urological cancer patients could be suitable for autologous cell therapy we studied the characteristics that ADSCs isolated from different non-oncogenic participants and cancer patients present upon expansion in xeno-free media and standard cell culture conditions. This study is to our knowledge the first to describe the successful isolation, expansion and characterization of ADSCs from urological neoplastic patients. We provide evidence for similar adequacy of *in vitro* expanded cancer patient derived ADSCs for autologous therapeutic purposes as shown by the equivalent phenotypical characteristics of their cells and EXOs.

Even though we are not aware of any previous study characterizing ADSCs from cancer patients, recent studies on bone marrow derived MSCs from breast cancer patients showed common morphology and regular growth ratios. Also, autologous transplantation results evidenced their safety [56]. Our results agree with theirs but in addition we provide a deeper characterization of patient derived MSCs in regard to their morphology, plasticity and paracrine potential. Furthermore, other studies showed that MSCs derived from non-carcinogenic pathologies such as multiple sclerosis presented similar characteristics to cells from non-oncogenic participants, such as cell proliferation, phenotype, *in vitro* differentiation and cytokine profile [57]. However, in both mentioned studies the cells evaluated were MSCs obtained from breast cancer patient bone marrow cells which require a more invasive method of obtaining MSCs; and neither of them used a xeno-free based cell expansion protocol that could be advantageous for the clinic.

Another important factor to be evaluated when optimization of cell therapy protocols is pursued is the relationship between patient age and expansion potential of their ADSCs. It has been previously shown that age negatively affects long-term expansion of MSCs from healthy donors *in vitro* [27], [29], [58]. Even though the number of patients evaluated in this study is very low to establish significance of the obtained values, the number of passages obtained in long term culture, was lower for ADSCs from the older patient (patient 2), coinciding with previous proposals that MSCs can be expanded longer before reaching senescence when isolated from younger donors. Nevertheless no significant differences in growth rates were observed for patients and non-oncogenic participants up to passage 4.

Limitation of cell expansion is directly related to replicative senescence in long-term cultures [59]. Our *in vitro* expanded ADSCs showed low β-galactosidase staining up to passage 4 but increased in later passages (Figure S1 A, B) which could possibly indicate limiting differentiation potential of our long term cultures as previously described [31], [60]. Another aging-associated marker we have characterized in early and late passages of cancer patient derived ADSCs is autophagy. The process of autophagy has been also proposed to be required for the maintenance of the unique properties of stem cells, namely pluripotency and differentiation, self-renewal and quiescence [34] and has been proposed as a marker of cell endurance which is desirable to favor engraftment of transplanted stem cells. In fact, a recent study has shown that survival of MSCs post-transplantation can be enhanced by drugs like atorvastatin, which activates autophagy via the AMPK/mTOR pathway, helping stem cells survive through post graft ischemic environment of hypoxia and serum deprivation, and evade apoptosis [61]. It is interesting to point out that atorvastatin (statin) an inhibitor of the 3-hydroxyl-3-methylglutaryl coenzyme A (HMG-CoA) reductase may exhibit anticancer effects as an autophagy inducer, since treatment with the statin showed cell killing and radiosensitizing effect of prostate cancer cells PC3 [62].

It is important to mention that the observed tendency in autophagosome structure formation at early passages, if ultrastructure images of cells at passage 4 are compared to those of cells at passage 2 (Figure 3 B–D), may indicate autophagosome induction in response to the stressing cell new environment and might be required for manteinance of the pluripotent state of the ADSCs. Being this the case induction of autophagosome formation on early passages could be interpreted as a marker for optimal cell endurance post-transplantation; and as shown by He *et al*., 2012 [62] adequate safety for cancer patient treatments. On the contrary, increased autophagy in late passages that show limited cell growth might indicate association to cell senescence and probably poor performance on transplantation. Cell senescence of our *in vitro* long term cultures is an indicative of non-being tumorigenic. No difference between autophagy structures or markers was appreciated in ADSCs derived from cancer patients and those from non-oncogenic participants (data not shown). In summary, no morphological differences, antigen surface profile or plasticity could be appreciated either in ADSCs derived from cancer patients with respect to those derived from non-oncogenic participants and therefore they could be considered as having an equivalent therapeutic potential for the clinic.

In contrast to the defined minimal criteria for expanded MSC [38], the sialomucin CD34 which is a hematopoietic stem cell marker, was expressed by a small fraction of our expanded ADSCs from both non-oncogenic participants and patients. This discrepancy may result from differences in the tissue used as the source of MSCs and to the xeno-free culture conditions we used in this study. In fact, a previous study by Suga *et al*., 2009 [39] shows that the CD34 marker is present in stem cells isolated from adipose tissue but its expression is gradually lost during expansion *in vitro*. Interestingly enough expression of the CD34 marker by ADSCs has been correlated with a more proliferative capacity, higher and increased expression of angiogenesis-related genes. In contrast, lack of CD34 correlate with greater ability for differentiation into adipogenic and osteogenic linages. Loss of CD34 marker has been correlated to time in culture [39], [63]. If the maintenance of certain proportion of CD34^+^ ADSCs in our cultures is consequence of the xeno-free medium used it would implicate that the *in vitro* expansion protocol described here might result advantageous when angiogenesis is pursued in the treatment.

With regard to the analysis of ADSCs derived EXOs we included in this study, two different perspectives should be taken into account. On one hand EXOs and their contents may provide themselves as effectors of the attributed beneficial paracrine effects of stem cell based therapies [13], [14], [16], [47]–[50]. In this sense we observed EXOs miRNA contents were comparable in EXOs from ADSCs isolated from cancer patients with those isolated from non-oncogenic participants, thus concluding similar EXO-mediated paracrine potential should be attributed to either ADSCs regardless of the health state of the donor.

However, on another hand, EXOs are associated with cross-talk tumor-related pathways such as epithelial-to-mesenchymal transition, cancer stemness and metastasis. The molecules mainly identified as mediators of the mentioned undesired features of the EXOs are miRNA [64]. Since the ADSCs we have studied derive from cancer patients it was necessary to characterize EXOs miRNA cargo in an attempt to reduce possible safety concerns. We did not observed differences between EXOs content in patients and non-oncogenic participants, at least for the miRNAs analyzed; suggesting ADSCs and their derivatives should be safe in this respect. We do not know whether these patients' blood or tissues contained tumorogenic cell-derived EXOs but expansion of ADSCs in culture should dilute them out.

It was worth noticing that some of the miRNAs previously shown to be expressed at altered levels in EXOs from cancer patients, as it was the case of miRNA let-7-a-1, miR-21, miR145, miR-451a and miR-1908 appeared at equivalent levels in EXOs from either patient or non-oncogenic participant ADSCs arguing in favor of their safe use in therapeutics. In particular, miR-21 overexpression had been observed in prostate cancer patients [65] coinciding with the type of cancer that some of the analyzed patients presented.

It is also important to point out that EXOs miRNA's cargo showed similar levels or were even enriched in EXOs with respect to ADSCs suggesting that in ADSCs some miRNAs are directionally targeted into EXOs before their release, as recently shown in other cell types [66]. It will be interesting to see whether sumoylation of heterogeneous nuclear ribonucleoprotein A2B1 or other hnRNP is required for the selection of the miRNAs to be packed into EXOs from MSCs as it has been shown in peripheral blood mononuclear cells (PBMCs) [66]. Being this the case sumoylation may represent a strategy to load stem cell derived EXOs with a particular cargo for therapeutic purposes.

Cancer patients carrying cancer-associated mutations in their genomes would not be candidates for autologous ADSC regenerative therapies. Furthermore, ADSCs derived from cancer patients with a normal karyotype could still be at risk of presenting cancer cells in their ADSC cultures. In order to evaluate this potential risk, a molecular karyotyping of the genome of expanded ADSCs from two of our participating patients was performed (Figure S3). Both patients presented a normal karyotype allowing us to assure that our methods for isolation and expansion of ADSC were appropriate for autologous cell therapy of these patients. Unfortunately, this does not guarantee that a safe fraction of ADSCs will be obtained from other patients under equivalent procedures.

MSC *in vitro* cell expansion has been shown to be safe under various cell culture conditions [67]–[69] the fact that we did not observed any alterations in the molecular karyotyping of the ADSCs from patients corroborates cell expansion under xeno-free conditions is safe for therapeutic purposes. However, we would like to recommend a molecular karyotype of the expanded ADSCs to be performed on a particular basis before autologous transplantation is approved. As an additional precaution molecular analysis of cancer patient ADSC derived EXOs content should be recommended to discard possible imbalances of their content that could compromised the safety of the transplanted cells.

Thus, since cancer patients usually are subjected to mutagenic procedures such as quimiotherapy or radiotherapy it should be preferable they bank their ADSCs before undergoing those procedures when autologous therapy is programmed.

Altogether these results suggest that autologous ADSCs provide a promising and safe strategy for cancer patient clinical cell therapy treatments.

## Conclusions

Our results demonstrate that ADSCs from cancer patients can be maintained under xeno-free culture conditions for the production of clinical-grade stem cells. Prior to recommend in vitro expanded ADSCs for autologous therapy in cancer patients molecular karyotyping and EXOs analysis are strongly recommended.

## Supporting Information

Figure S1 **Senescence of** ***in vitro*** **expanded ADSCs from cancer patients and non-oncogenic participants.** Senescence associated β-galactosidase activity was detected in late passages of *in vitro* expanded ADSCs (B) in reference to early passages (A) (phase contrast microscopy images at 20X). Relative expression of autophagy related genes were determined by RT-PCR in early *vs* late passages (C). Lanes 1–2 GAPDH, 3–4 Atg5, 5–6 Atg7 and 7–8 Beclin 1. Abundant autophagic structures appeared in late passages as shown by electron microscopy images (arrows). Scale bar 20 µm (D); 2 µm (E).
(TIF)

Figure S2 **ADSC and EXO miRNA expression levels from cancer patients and non-oncogenic participants.** Total RNA isolated from ADSCs and ADSC-derived EXOs of patients (A) and non-oncogenic participants (B) was tested for the expression of selected miRNAs by qRT-PCR; Ct values are shown.
(TIF)

Figure S3 **Molecular Karyotype of** ***in vitro*** **expanded ADSCs from cancer patients.** Cancer patients 1 and 5 ADSC passage 4 genomes were analyzed. Array results revealed only polymorphic gains of 280 kb in chromosome 6 and 578 kb in chromosome 16 for patient 1 (A) and a polymorphic loss of 116 kb in chromosome Y for Patient 5 (B). These findings confirm the safety of *in vitro* expanded ADSCs from cancer patients.
(TIF)

Figure S4 **Expression of ADSC surface markers from donor cells.** Representative flow fluorescence activated cell sorting (FACS) of *in vitro* expanded ADSCs from donors at passage 4. Cells were positive for CD105 (A), CD73 (B), CD90 (C) but do not express CD11b (D); while CD34 was partially positive (E), as previously described. Panel F shows isotypes IgG1 and IgG3 cytometric analysis.
(TIF)

Table S1 **miRNAs studied on ADSCs and ADSCs-derived Exosomes (√ evidenced; - not evidenced).**
(DOCX)
