# Supplementary figures and images for: Therapeutic Potential of Human Adipose-Derived Stem Cells (ADSCs) from Cancer Patients: A Pilot Study
Source: PLoS One. 2014 Nov 20;9(11):e113288. doi: 10.1371/journal.pone.0113288 (PMC4239050; doi:10.1371/journal.pone.0113288)

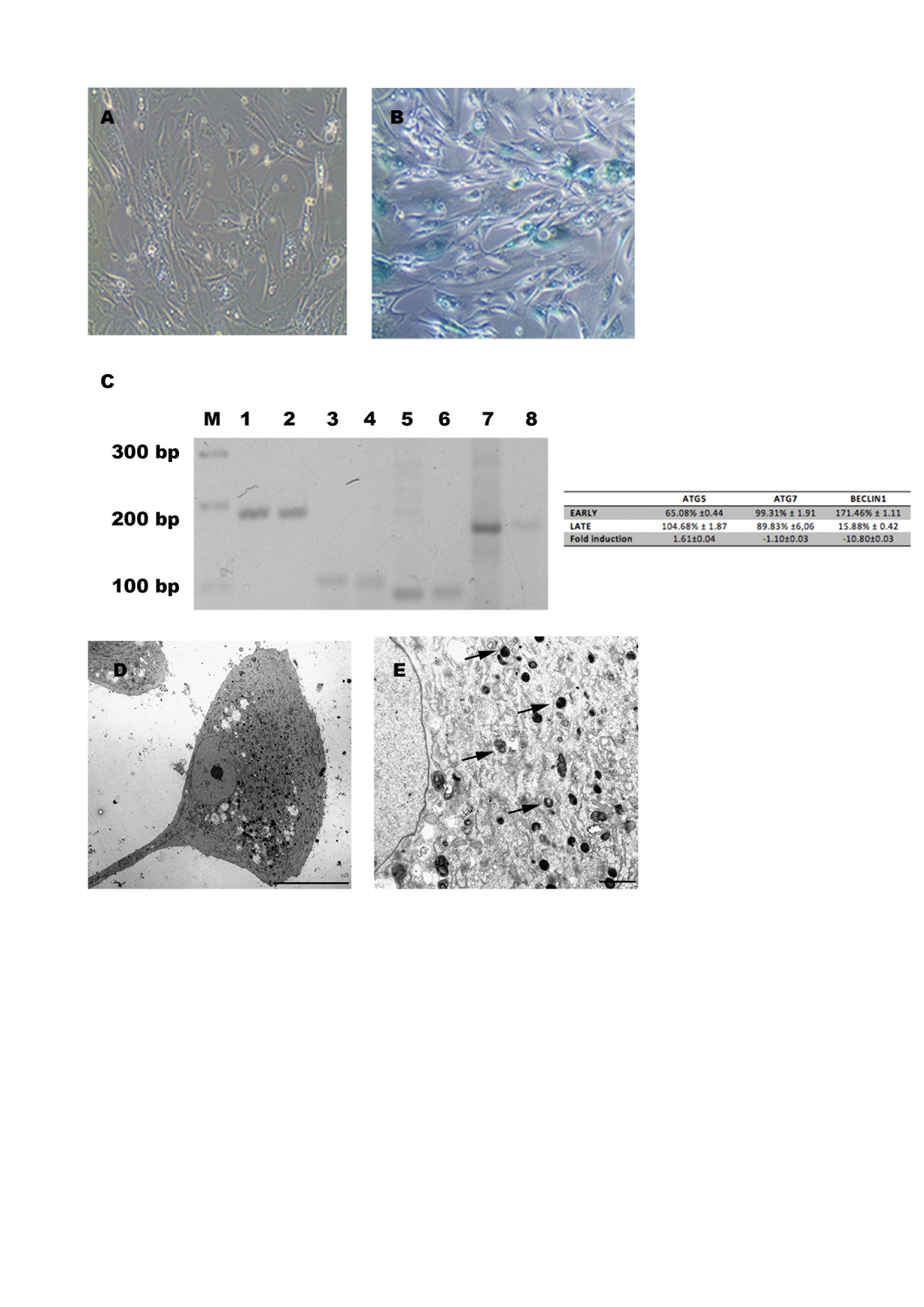

Supplement: Figure S1 — Senescence of in vitro expanded ADSCs from cancer patients and non-oncogenic participants. Senescence associated β-galactosidase activity was detected in late passages of in vitro expanded ADSCs (B) in reference to early passages (A) (phase contrast microscopy images at 20X). Relative expression of autophagy related genes were determined by RT-PCR in early vs late passages (C). Lanes 1–2 GAPDH, 3–4 Atg5, 5–6 Atg7 and 7–8 Beclin 1. Abundant autophagic structures appeared in late passages as shown by electron microscopy images (arrows). Scale bar 20 µm (D); 2 µm (E). (TIF) [file pone.0113288.s001.tif]

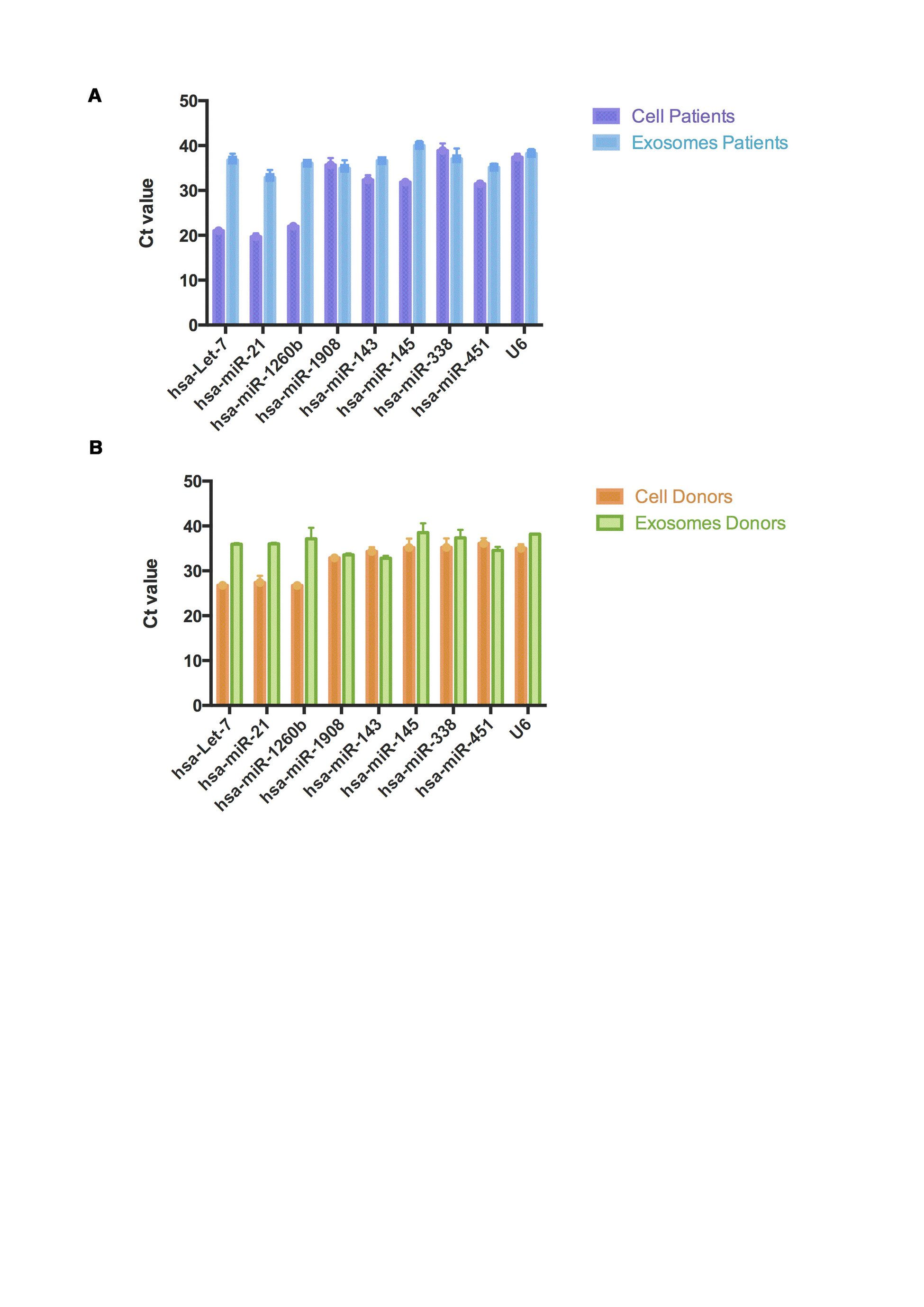

Supplement: Figure S2 — ADSC and EXO miRNA expression levels from cancer patients and non-oncogenic participants. Total RNA isolated from ADSCs and ADSC-derived EXOs of patients (A) and non-oncogenic participants (B) was tested for the expression of selected miRNAs by qRT-PCR; Ct values are shown. (TIF) [file pone.0113288.s002.tif]

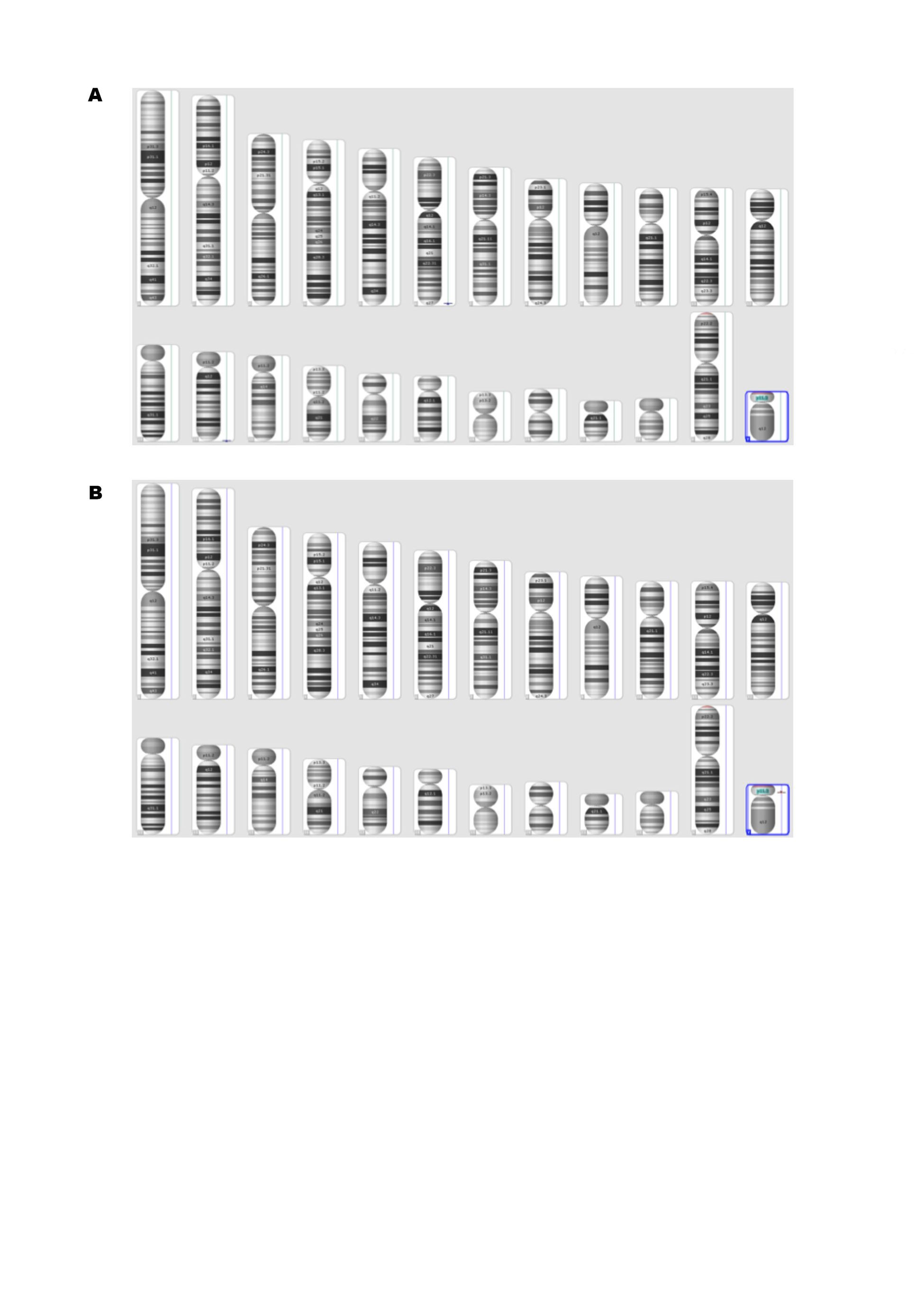

Supplement: Figure S3 — Molecular Karyotype of in vitro expanded ADSCs from cancer patients. Cancer patients 1 and 5 ADSC passage 4 genomes were analyzed. Array results revealed only polymorphic gains of 280 kb in chromosome 6 and 578 kb in chromosome 16 for patient 1 (A) and a polymorphic loss of 116 kb in chromosome Y for Patient 5 (B). These findings confirm the safety of in vitro expanded ADSCs from cancer patients. (TIF) [file pone.0113288.s003.tif]

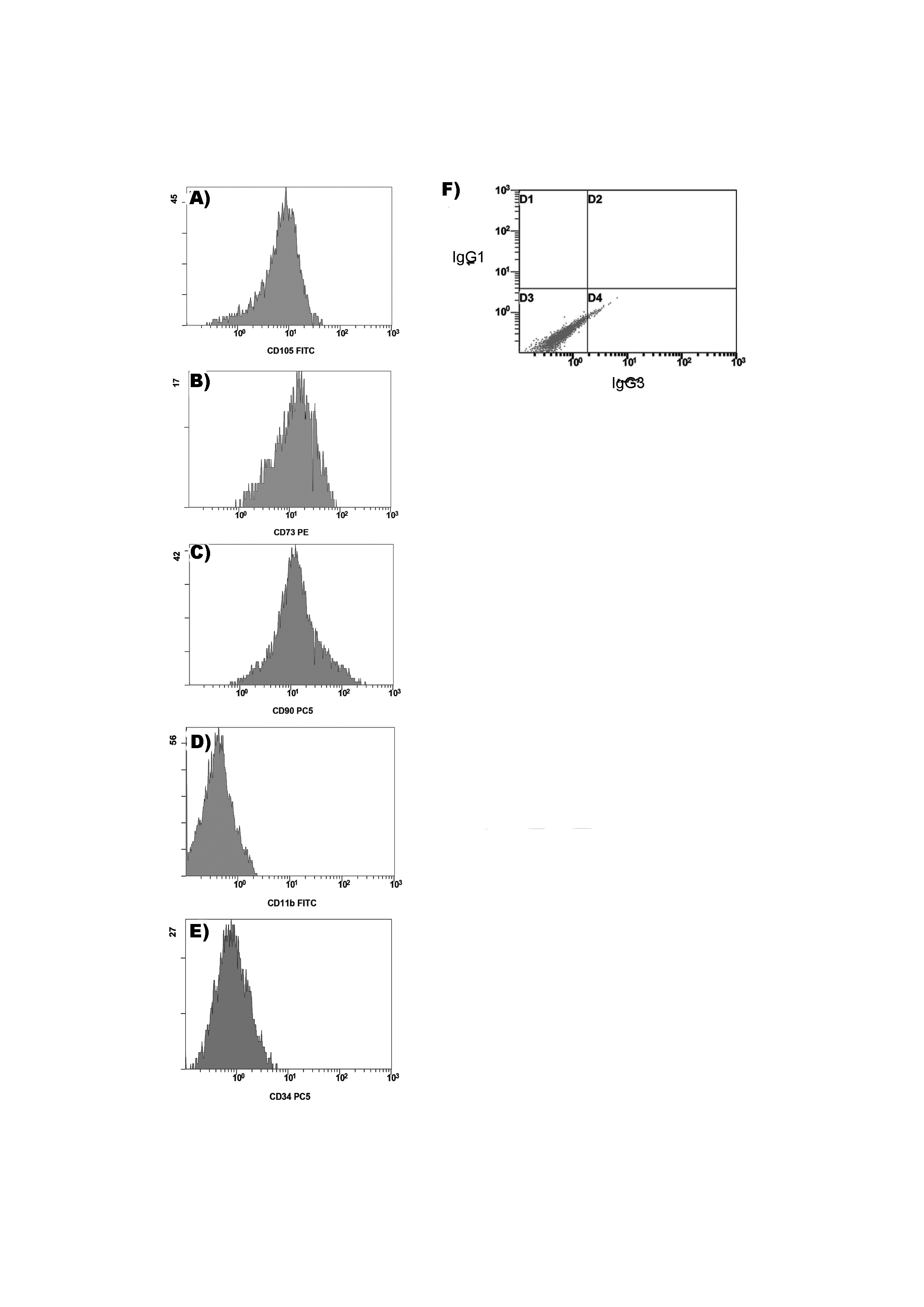

Supplement: Figure S4 — Expression of ADSC surface markers from donor cells. Representative flow fluorescence activated cell sorting (FACS) of in vitro expanded ADSCs from donors at passage 4. Cells were positive for CD105 (A), CD73 (B), CD90 (C) but do not express CD11b (D); while CD34 was partially positive (E), as previously described. Panel F shows isotypes IgG1 and IgG3 cytometric analysis. (TIF) [file pone.0113288.s004.tif]
