# Supplementary material for: Therapeutic Potential of Human Adipose-Derived Stem Cells (ADSCs) from Cancer Patients: A Pilot Study
Source: PLoS One. 2014 Nov 20;9(11):e113288. doi: 10.1371/journal.pone.0113288 (PMC4239050; doi:10.1371/journal.pone.0113288)
Supplement: Table S1 — miRNAs studied on ADSCs and ADSCs-derived Exosomes (√ evidenced; - not evidenced). (DOCX) [file pone.0113288.s005.docx]

| Name | MSCs | MSC EXOs | Cancer | Cancer EXOs | References |
| --- | --- | --- | --- | --- | --- |
| hsa-let-7a-1 | √ | √ | √ | √ | 52,I-III |
| hsa-miR-21 | √ | √ | √ | √ | 51,52,I,IV |
| hsa-miR-1260b | - | - | √ | - | V,VI |
| hsa-miR-1908 | - | - | √ | √ | VII |
| hsa-miR-143 | √ | √ | √ | - | 52,VIII-X |
| hsa-miR-145 | √ | √ | √ | √ | 52,IV,VIII,IX,XI |
| hsa-miR-338-3p | - | - | √ | √ | XII-XV |
| hsa-miR-451a | √ | √ | √ | √ | 51,56,IV,XVI |

**Supplementary References**

1. Rana S, Malinowska K, Zöller M (2013) Exosomal tumor microRNA modulates premetastatic organ cells. Neoplasia 15(3): 281-95.
2. Ohshima K, Inoue K, Fujiwara A, Hatakeyama K, Kanto K *et al*. (2010) Let-7 microRNA family is selectively secreted into the extracellular environment via exosomes in a metastatic gastric cancer cell line. PLoS One 5(10): e13247.
3. Yang Q, Jie Z, Cao H, Greenlee AR, Yang C *et al*. (2011) Low-level expression of let-7a in gastric cancer and its involvement in tumorigenesis by targeting RAB40C. Carcinogenesis 32(5): 713-22.
4. To KK (2013) MicroRNA: a prognostic biomarker and a possible druggable target for circumventing multidrug resistance in cancer chemotherapy. J Biomed Sci. 20:99.
5. Hirata H, Hinoda Y, Shahryari V, Deng G, Tanaka Y *et al.* (2014) Genistein downregulates onco-miR-1260b and upregulates sFRP1 and Smad4 via demethylation and histone modification in prostate cancer cells. Br J Cancer 110(6): 1645-54.
6. Hirata H, Ueno K, Nakajima K, Tabatabai ZL, Hinoda *Y et al.* (2013) Genistein downregulates onco-miR-1260b and inhibits Wnt-signalling in renal cancer cells. Br J Cancer 108(10): 2070-8.
7. Feng DQ, Huang B, Li J, Liu J, Chen XM *et al*. (2013) Selective miRNA expression profile in chronic myeloid leukemia K562 cell-derived exosomes. Asian Pac J Cancer Prev 14(12): 7501-8.
8. Guo L, Zhao RC, Wu Y (2011) The role of microRNAs in self-renewal and differentiation of mesenchymal stem cells. Exp Hematol 39(6): 608-16.
9. Michael MZ, O' Connor SM, van Holst Pellekaan NG, Young GP, James RJ (2003) Reduced accumulation of specific microRNAs in colorectal neoplasia. Mol Cancer Res 1(12): 882-91.
10. Zhang Y, Wang Z, Chen M, Peng L, Wang X *et al.* (2012) MicroRNA-143 targets MACC1 to inhibit cell invasion and migration in colorectal cancer. Mol Cancer 11: 23.
11. Akao Y, Khoo F, Kumazaki M, Shinohara H, Miki K *et al.* (2014) Extracellular Disposal of Tumor-Suppressor miRs-145 and -34a via Microvesicles and 5-FU Resistance of Human Colon Cancer Cells. Int J Mol Sci 15(1): 1392–1401.
12. Xue Q, Sun K, Deng HJ, Lei ST, Dong JQ *et al*. (2014) MicroRNA-338-3p inhibits colorectal carcinoma cell invasion and migration by targeting smoothened. Jpn J Clin Oncol 44(1):13-21.
13. Huang XH, Chen JS, Wang Q, Chen XL, Wen L, Chen LZ, Bi J, Zhang LJ, Su Q, Zeng WT (2011) miR-338-3p suppresses invasion of liver cancer cell by targeting smoothened. J Pathol 225(3): 463-72.
14. Wenying Li, Wei Xiong, Xiaomei Chen, Wei Liu, Shiang Huang *et al*. (2013) Oncomirnas and tumor suppressors in microvesicles from four types of cancer. Blood 122(2): 4900.
15. Kozubek J, Ma Z, Fleming E, Duggan T, Wu R *et al.* (2013) In-depth characterization of microRNA transcriptome in melanoma. PLoS One 8(9): e72699.
16. Pigati L, Yaddanapudi SC, Iyengar R, Kim DJ, Hearn SA *et al*. (2010) Selective release of microRNA species from normal and malignant mammary epithelial cells. PLoS One 25(10): e13515.
